# Unveiling the role of copper metabolism and STEAP2 in idiopathic pulmonary fibrosis molecular landscape

**DOI:** 10.1111/jcmm.18414

**Published:** 2024-06-14

**Authors:** Yajun Wang, Shuyang Chen, Shujing Chen, Jinjun Jiang

**Affiliations:** ^1^ Department of Pulmonary and Critical Care Medicine, Zhongshan Hospital Fudan University Shanghai China; ^2^ Shanghai Respiratory Research Institute Shanghai China

**Keywords:** bioinformatics, copper metabolism, IPF, STEAP2, WGCNA

## Abstract

Idiopathic pulmonary fibrosis (IPF) is a debilitating interstitial lung disease characterized by progressive fibrosis and poor prognosis. Despite advancements in treatment, the pathophysiological mechanisms of IPF remain elusive. Herein, we conducted an integrated bioinformatics analysis combining clinical data and carried out experimental validations to unveil the intricate molecular mechanism of IPF. Leveraging three IPF datasets, we identified 817 upregulated and 560 downregulated differentially expressed genes (DEGs). Of these, 14 DEGs associated with copper metabolism were identified, shedding light on the potential involvement of disrupted copper metabolism in IPF progression. Immune infiltration analysis revealed dysregulated immune cell infiltration in IPF, with a notable correlation between copper metabolism‐related genes and immune cells. Weighted gene co‐expression network analysis (WGCNA) identified a central module correlated with IPF‐associated genes, among which STEAP2 emerged as a key hub gene. Subsequent in vivo and in vitro studies confirmed the upregulation of STEAP2 in IPF model. Knockdown of STEAP2 using siRNA alleviated fibrosis in vitro, suggesting potential pathway related to copper metabolism in the pathophysiological progression of IPF. Our study established a novel link between immune cell infiltration and dysregulated copper metabolism. The revelation of intracellular copper overload and upregulated STEAP2 unravelled a potential therapeutic option. These findings offer valuable insights for future research and therapeutic interventions targeting STEAP2 and associated pathways in IPF.

## 1 INTRODUCTION

Idiopathic pulmonary fibrosis (IPF) is a chronic and progressive interstitial lung disease of unknown aetiology, characterized by an unfavourable prognosis.^1^, ^2^ Epidemiological studies on IPF indicate a global incidence ranging from 0.09 to 1.30 per 10,000 people, with a steady annual increment.^3^ The histopathological manifestations of IPF encompass the abnormal accumulation of extracellular matrix (ECM) in alveoli, leading to the distortion of normal lung architecture and eventually irreversible impairment of pulmonary function.^4^ Previous studies have identified chronic inflammation to be the pathological basis of fibrosis. Clinical presentations of IPF are characterized by a gradual onset of breathlessness and a pronounced reduction in lung compliance.^3^ Despite the availability of antifibrotic medications, the median survival generally does not exceed 5–10 years after diagnosis.^5^

Copper (Cu) is essential for various biological processes, functioning not only as a catalyst and an antioxidant, but also a promoter of autophagy and even an initiator of immune response.^6^ The inherent ability of oxidation–reduction renders Cu both advantageous and potentially detrimental to cells.^7^ *Tsvetkov* et al. demonstrated that Elesclomol, a Cu ionophore, causes intracellular Cu overload and subsequently toxic aggregation of lipoylated proteins and destabilization of Fe‐S cluster proteins, which leads to elevated proteotoxic stress and the eventual death of cells. Furthermore, it was demonstrated that cells with active mitochondrial activities are more susceptible to Cu toxicity.^8^ Interestingly, mitochondrial dysfunction is a crucial phenomenon in the pathogenesis of IPF.^9^, ^10^ Previous bioinformatic analysis revealed certain correlations between Cuproptosis‐related genes (CRGs) and outcomes of IPF.^11^ Intracellular Cu homeostasis plays a pivotal role in the differentiation of immune cells and functional human immunity.^12^ Genes responsible for maintaining metabolism (e.g. NLRP3) have been discovered to also participate in TGF‐β and EMT signalling pathways, suggesting contributory roles in the exacerbation of fibrosis.^13^

We attempted to elucidate the correlation between CRGs expressions and immune infiltration. Datasets of IPF were acquired from gene expression omnibus (GEO) database. After identifying differentially expressed genes (DEGs), WGCNA was used to identify genes of significance within modules. STEAP2 was identified as a key hub gene. As a member of STEAP family, STEAP2 stimulated cellular uptake of both iron and Cu in vitro.^14^ Subsequent in vivo and in vitro confirmed the upregulation of STEAP2 in IPF. Knockdown of STEAP2 ameliorated fibrosis and the pathogenesis of IPF.

## 2 METHODS AND MATERIALS

### 2.1 Data acquisition

‘IPF’ was used to search targeted datasets. GSE110147,^15^ GSE24206,^16^ GSE53845^17^ were obtained from the GEO database, comprising lung tissue from 25 healthy controls and 73 IPF cases.

### 2.2 Data processing and DEGs identification

Using the R package ‘affy’, we performed background calibration, normalization, and log2 transformation on the IPF datasets. If multiple probes identified the same gene, we calculated the average value to represent its expression. After combining the three datasets, ‘SVA’ package was used to remove batch effects. ‘Limma’ package was employed to identify DEGs with a cutoff value of *p* >0.05 and Fold change (FC) >1.5.

### 2.3 Immune infiltration analysis

We employed ‘CIBERSORT’ package to calculate the scores representing the infiltration of immune cells in each individual sample.^18^ The vioplotwas used to demonstrate the variations in the proportions of different immune cell types between the IPF and control groups. ‘corrplot’ package was used to visualize the correlation among the infiltrating immune cells.

### 2.4 Protein–Protein Interaction (PPI) networks construction

STRING was used to construct a PPI network of DEGs The String database was used to construct a PPI network of DEGs related to Cu metabolism.^19^ Cytospace and its MCODE plugin was used to visualize the interaction between said proteins.

### 2.5 Functional enrichment analysis

We employed the ‘clusterProfiler’ R package for functional enrichment analysis, encompassing gene ontology (GO) and Kyoto Encyclopedia of Genes and Genomes (KEGG) analysis.^20^

### 2.6 Weighted gene co‐expression network analysis (WGCNA)

WGCNA algorithm was used to construct gene clustering modules. A gene co‐expression network of genes from normal lung samples and IPF samples was generated using ‘WGCNA’ R package.^21^ We first calculated and selected an optimal soft‐thresholding power and construct the adjacency matrix based on the selected power threshold. Next, hierarchical clustering tree was established to cluster high co‐expression genes into the same module. Finally, immune cell infiltration, CRGs were used as characteristics to compute the correlation between module genes and traits. Sangerbox was used to visualize the gene expression network.

### 2.7 LASSO logistic regression

LASSO analysis was employed for the optimal feature selection in high‐dimensional data to identify hub genes associated with Cu metabolism in IPF. The model was constructed using the ‘glmnet’ R package, demonstrating its capacity to differentiate between IPF patients and controls.

### 2.8 Animal model

IPF mouse model was constructed in male C57BL/6 mice aged 8–10 weeks by intratracheally administering Bleomycin (Cat. No. HY‐17565A, MCE, China, 5 mg/Kg) dissolved in saline. Mice were housed in a controlled environment with ad libitum access to food and water. All animal procedures and protocols were approved by the Ethics Committee of Zhongshan Hospital, Fudan University.

### 2.9 Histology analysis

Paraffin‐embedded lung sections (4 μm) were prepared according to standard protocols. Haematoxylin and Eosin (H&E) staining and Masson staining were performed. Sections and then stained using Haematoxylin and Eosin. Independent pathologists unaware of the sources of the samples were invited to score the slides following the Immuno‐Reactive Score (IRS) system, three sections were selected to be examined from each sample. The resulting average of these scores was documented as the ultimate lung tissue damage score and IRS.

### 2.10 Real‐time quantitative PCR (RT‐qPCR)

Total RNA was extracted using RNA extraction kit following the manufacturer's protocol (Cat. No. B618203, Sangon Biotech, Shanghai). Extracted RNA served as the template for cDNA synthesis employing the PrimeScript™ RT reagent Kit (Cat. No. RR037A, Takara Bio, Japan). Quantitative reverse transcription polymerase chain reaction (RT‐qPCR) was performed using SYBR® Green (Cat. No. 11201ES03, Yeason, Shanghai) on a Step One Plus Real‐Time PCR System (Quantstudio 5, Thermo Fisher). Gene expression levels were quantified and analysed utilizing the 2^‐△△CT method.

GAPDH F 5’‐TGGCCTTCCGTGTTCCTAC‐3’.

GAPDH R 5’‐GAGTTGCTGTTGAAGTCGCA‐3’.

STEAP2 F 5’‐ATGGGAAGCCCTAAGAGCCT‐3’.

STEAP2 R 5’‐GTGGTAGCCACATCTAATAAGCC‐3′.

### 2.11 Western blotting

Lung tissues were lysed using RIPA buffer (Cat. No. P0013B, Beyotime, China) supplemented with phosphatase (Cat. No. P1045, Beyotime, China) and protease inhibitors (Cat. No. P1005, Beyotime, China). Protein concentrations were determined using the Braford assay. Proteins were separated via SDS‐PAGE in each experiment. Following SDS‐PAGE, the proteins were transferred onto PVDF membranes and incubated with antibodies (STEAP2: Cat. No. 20201‐1‐AP; Proteintech, Wuhan; TUBULIN: Cat. No. abs830032, Abcam, Shanghai) overnight at 4°C. Following the TBST wash, the membranes were subjected to HRP‐conjugated secondary antibodies for a duration of 60 min. ECL detection reagents were used for visualization of the results. The obtained outcomes were analysed using Image J version V1.8.0.112 (NIH).

### 2.12 Cell line and SiRNA interference

MRC5 cell lines were obtained from the American Type Culture Collection (Shanghai Fuheng Biology Co.). Cells had been proven to be free of mycoplasma contamination. MRC5 cells were maintained in Dulbecco's Modified Eagle Medium (DMEM, Cat. No. 11965092, Gibco, United States) with 10% Fetal Bovine Serum (FBS, Cat. No. F0193, Sigma‐Aldrich, Uniter States) and 1100 U/mL of penicillin, and 100 μg/mL of streptomycin at 37°C and 5% CO2. MRC5 cells were transfected with siRNA (40 μM) using Lipofectamine 2000 (Cat. No. 18324012, Invitrogen, United States) following manufacturer's instructions. The siRNA targeting STEAP2 were synthesized by Hanbio (Shanghai). Sequences were as follows:

STEAP2‐si‐1 sense 5’‐CCUAAUGGCAUAAAUGGUATT‐3’.

STEAP2‐si‐1 antisense 5’‐UACCAUUUAUGCCAUUAGGTT‐3’.

STEAP2‐si‐2 sense 5’‐CAGAAUCCAAUGCUGAAUATT‐3’.

STEAP2‐si‐2 antisense 5’‐UAUUCAGCAUUGGAUUCUGTT‐3’.

STEAP2‐si‐3 sense 5’‐GAUUUUAUACACCACCAAATT‐3’.

STEAP2‐si‐3 antisense 5’‐UUUGGUGGUGUAUAAAAUCTT‐3’.

Negative Control (NC) sense 5’‐UUCUCCGAACGUGUCACGUTT‐3’.

NC antisense 5’‐ACGUGACACGUUCGGAGAA‐3′.

### 2.13 Statistical analysis

The continuous variables were reported as the means ± SEM and compared using Student's t‐test. Data were analysed using GraphPad Prism Version 8.3.0 (GraphPad Software, San Diego, CA, USA). *p* value <0.05 was considered to be statistically significant.

## 3 RESULTS

### 3.1 Identification of DEGs

**FIGURE 1:**
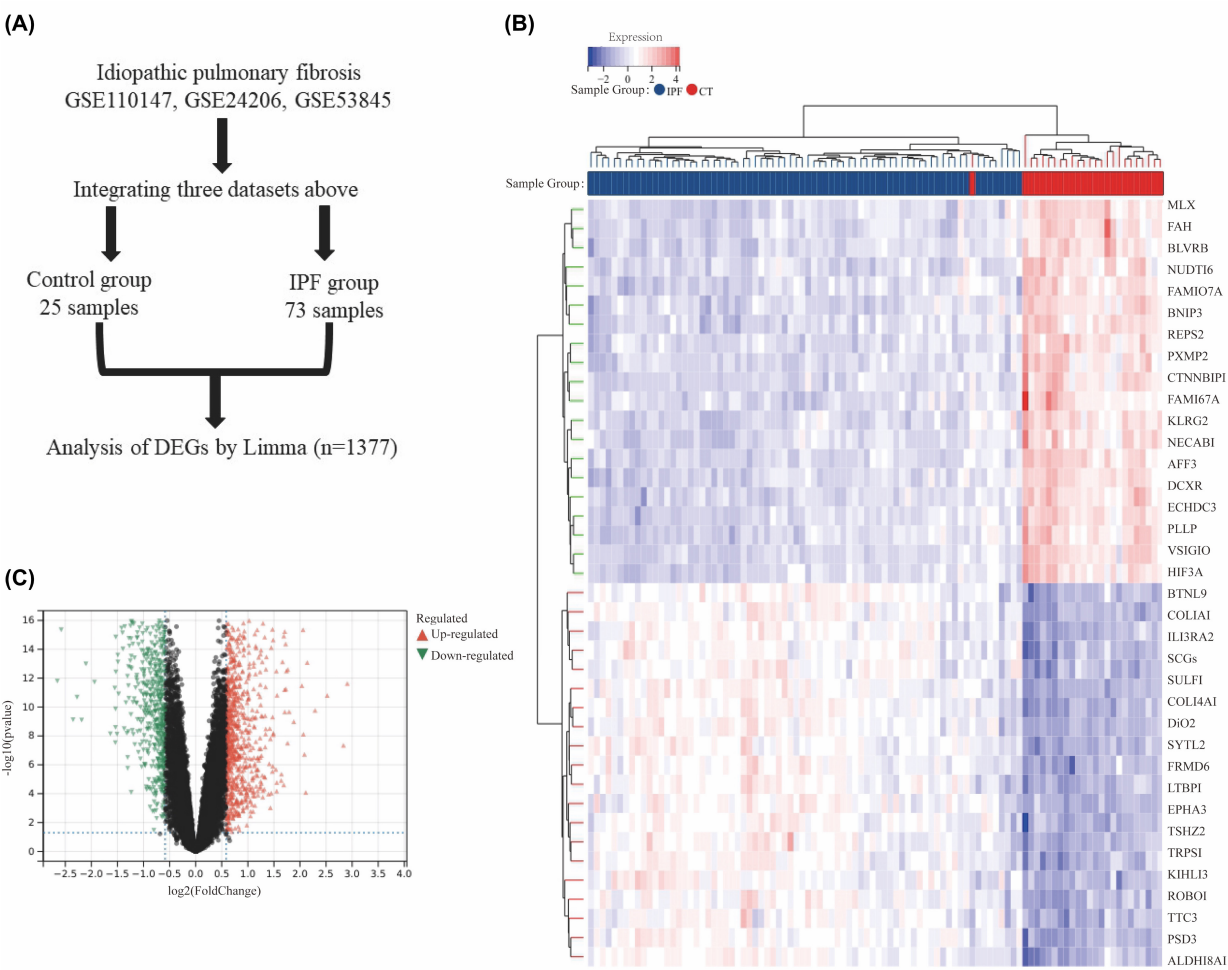
Analysis of Differentially Expressed Genes (DEGs) in the Integrated Idiopathic Pulmonary Fibrosis (IPF) Dataset. (A) Flowchart illustrating the merging of three IPF datasets. (B) Heatmap displaying rows corresponding to DEGs and columns representing samples from either IPF cases or controls. (C) Volcano plot highlighting DEGs, with triangles in red and green indicating upregulated and downregulated gene expression, respectively.

Three IPF datasets were merged and normalized to remove batch differences (Figure 1A). Limma algorithm was employed to identify DEGs. Of the DEGs identified, 817 were upregulated, while 560 genes were downregulated. The heatmap and volcano plot depicting the DEGs in IPF are presented in Figure 1B,C.

### 3.2 Expression profile of cuproptosis and Cu metabolism‐associated genes in IPF

**FIGURE 2:**
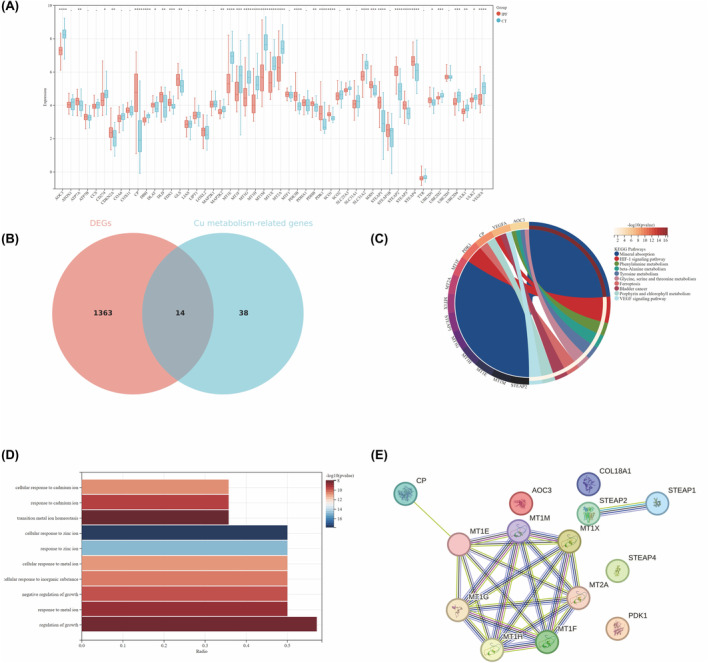
Expression and Analysis of Copper Metabolism‐Associated Genes in Idiopathic Pulmonary Fibrosis (IPF). (A) Comparison of copper metabolism‐related gene expression between IPF and control groups. (B) Venn diagram showing five genes shared among copper metabolism‐related genes. (C) KEGG pathway analysis of the 14 common genes. (D) Gene Ontology (GO) analysis of the 14 common genes. (E) Protein‐Protein Interaction (PPI) network of the 14 common genes.

Our study focus on the differential expression of 52 genes associated with cuproptosis and Cu metabolism^22^ between IPF and control groups (Figure 2A). In the IPF group (Figure 2B), a total of 14 DEGs were identified. In summary, these findings underscore the significant role of Cu metabolism‐related genes in the development of IPF. Further investigation into the interrelationships among the 14 Cu metabolism‐related DEGs, using KEGG enrichment analysis (Figure 2C), revealed that these genes are primarily involved in the pathways of mineral absorption and HIF‐1 signalling. The subsequent GO (Figure 2D) analysis indicated a substantial enrichment of genes associated with the regulation of growth. All of the signalling pathways and ontologies mentioned above were vital for the pathogenesis of IPF. Additionally, the PPI network illustrates interactions among 14 genes (Figure 2E), with positive correlation between members of metallothioneins (MTs) family and 6‐transmembrane epithelial antigen of prostate (STEAP) family interact positively.

### 3.3 Immune cell infiltration analysis and Lasso‐Cox regression

**FIGURE 3:**
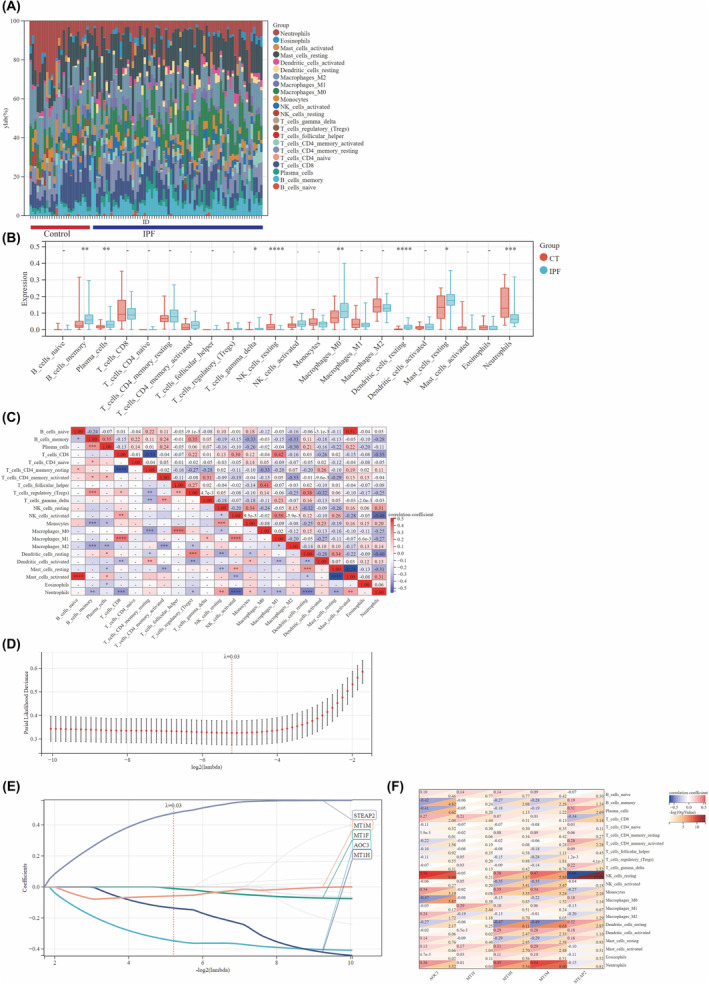
Immune Infiltration Analysis and Lasso‐Cox Regression. (A) This barplot displays the proportions of various immune cell types across different samples. (B) A violin plot compares the proportions of immune cell types between the IPF and control groups. (C) Correlation analysis is shown for the compositions of different immune cell types. (D) Selection of the tuning parameter (lambda) in the LASSO regression model. (E) Profiles of LASSO coefficients. (F) Correlation analysis between immune cell types and five copper metabolism‐related differentially expressed genes (DEGs). Significance levels are indicated as *p < 0.05; **p < 0.01.

Immune cell infiltration scores for each sample were computed using the ‘CIBERSORT’ R package, with the barplot for the IPF and control groups visually represents the distribution of 22 immune cell types (Figure 3A). The vioplot (Figure 3B) illustrated that IPF samples exhibited an elevated level of memory B cells, plasma cells, gamma delta T cells, M0 macrophages, resting dendritic cells and resting mast cells, alongside reduced levels of resting NK cells and neutrophils. Correlation analysis among the 22 immune cell types revealed a positive association between naive B cells and activated mast cells (*r* = 0.51), while resting natural killer cells showed a negative correlation with neutrophils (*r* = −0.46) (Figure 3C). To uncover hub genes related to the Cu metabolism in IPF, the LASSO model was constructed using the expression levels of Cu metabolism related‐genes and clinical traits (IPF vs. control) across all samples (Figure 3D). According to the value of the lambda minimum criteria, five genes were identified to have nonzero regression coefficients (Figure 3E). We also analysed the correlation between the five DEGs related to Cu metabolism and immune infiltration. The heatmap (Figure 3F) revealed a positive correlation between resting NK cells and AOC3 (*r* = 0.56), a positive association between neutrophils and MT1M (*r* = 0.56), while resting NK cells exhibited a negative correlation with STEAP2 (*r* = 0.66).

### 3.4 WGCNA and identification of key modules

**FIGURE 4:**
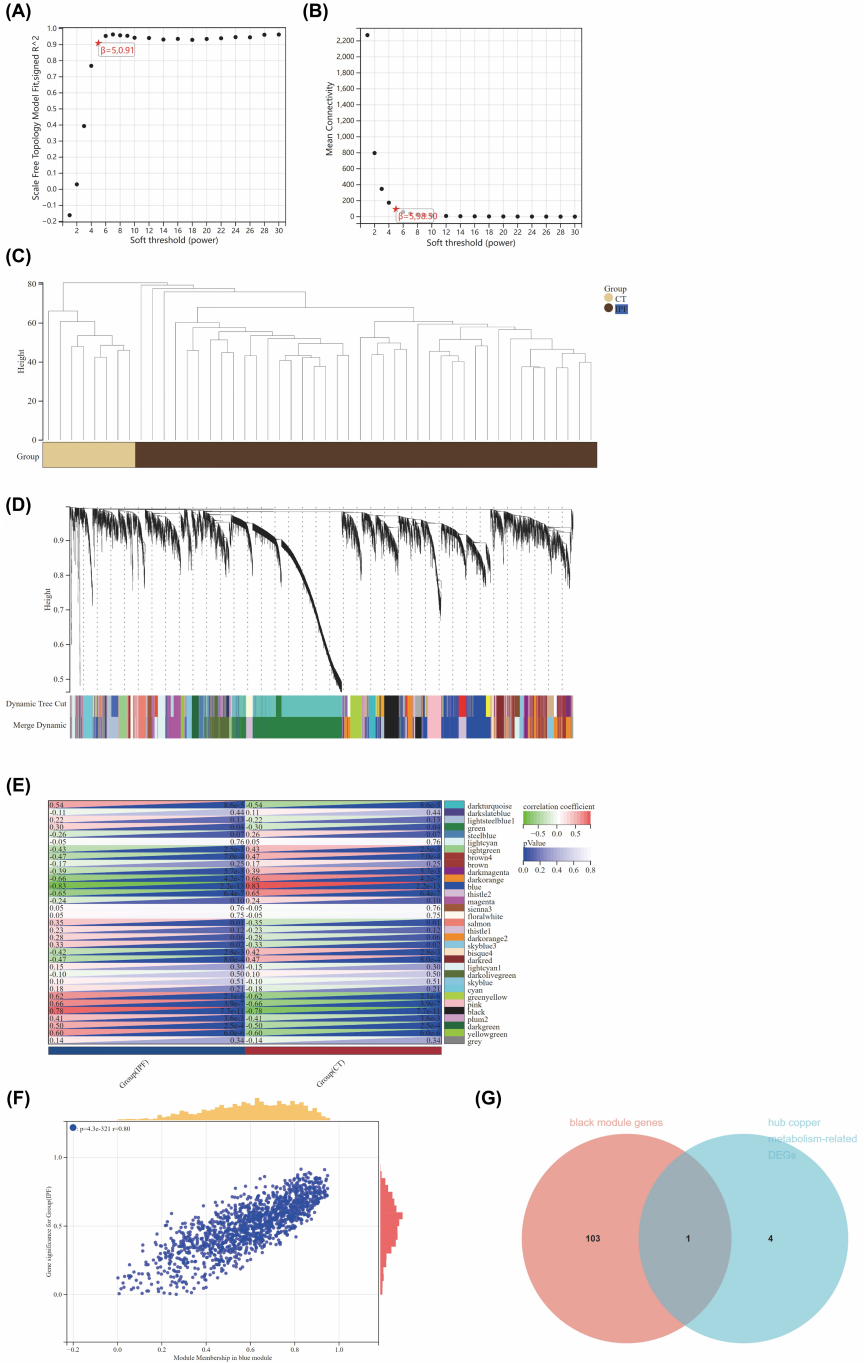
WGCNA in IPF. (A) Assignment of a soft threshold of 5 through comprehensive assessment. (B) Reiteration of a soft threshold of 5 designated after a further comprehensive assessment. (C) Visualization of a clustering dendrogram for all samples. (D) Identification of distinct gene co‐expression modules, each highlighted in different colors on the gene tree. (E) Analysis of the relationship between gene modules and IPF, with the black module showing significant correlation. (F) Examination of the relationship between module membership and gene significance within the black module. (G) Identification of a shared gene between hub genes related to copper metabolism and genes in the black module.

A single IPF dataset (GSE53845) was subjected to WGCNA, with a ‘soft’ thresholding power of *β* = 5 (achieving a scale‐free R^2^ of 0.91) as shown in Figure 4A,B. Figure 4C displays the clustering dendrogram of IPF and control samples. Subsequently, 33 distinct gene co‐expression modules (GCMs) were identified, each designated by a unique colour (Figure 4D). Figure 4E illustrated the association between IPF and these GCMs. Significantly, the black module, consisting of 104 genes, displayed the strongest association with IPF (correlation coefficient = 0.78, *p* = 7.7 × 10^−11^) and was recognized as the central module for further analysis. Moreover, we calculated the associations between module affiliation and gene importance in the black module for IPF. As expected, there was a clear and significant positive correlation between them (*r* = 0.69), as shown in Figure 4F. As a result, the genes in the black module were determined to have the strongest association with IPF. In Figure 4G, the identification of 1 gene (STEAP2) occurred by intersecting 5 hub DEGs related to Cu metabolism with 104 genes in the black module.

### 3.5 STEAP2 expression in animal model

**FIGURE 5:**
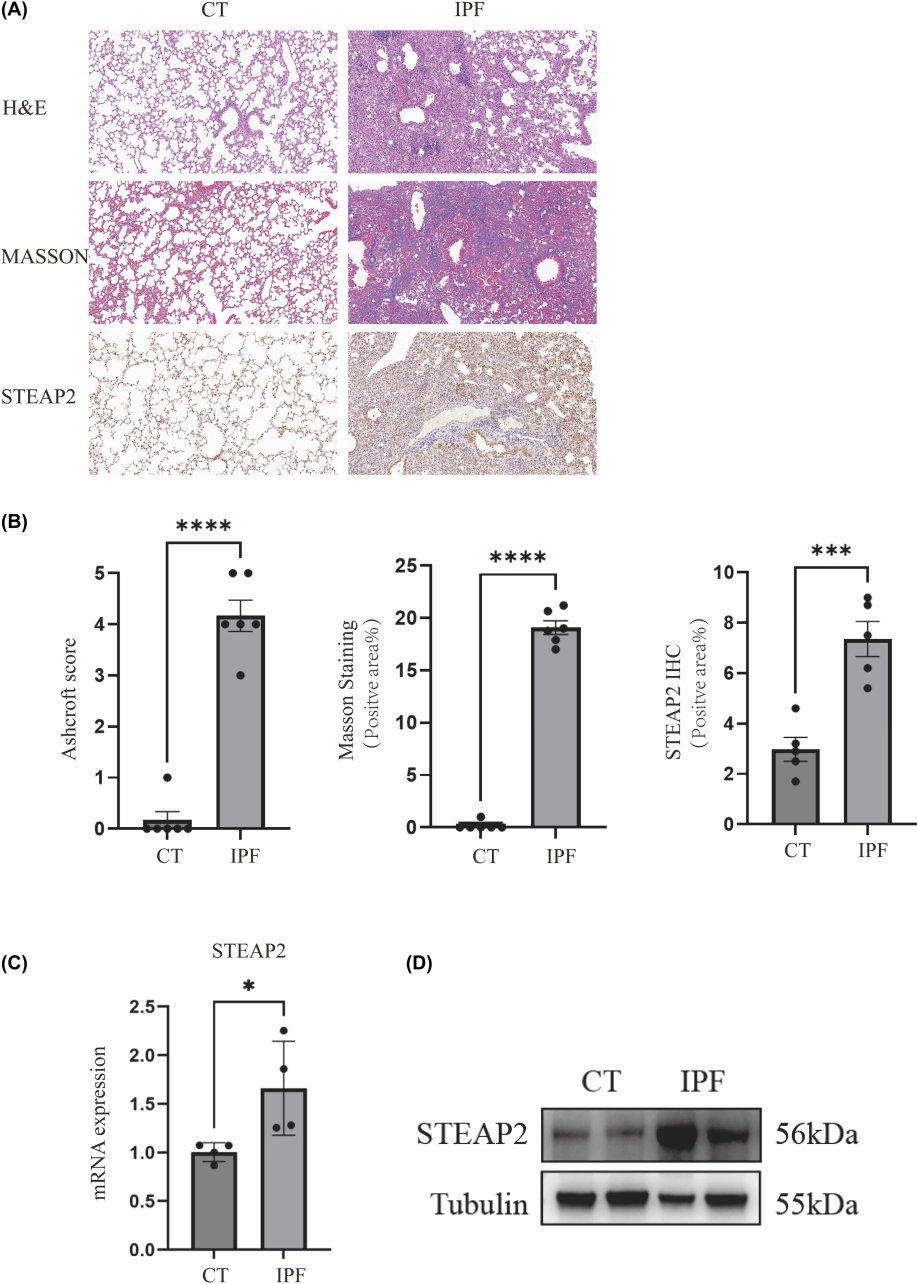
H&E, Masson, STEAP2 Immunohistochemical Staining, and STEAP2 Expression in Western Blot and RT‐qPCR. (A) H&E, Masson’s trichrome, and STEAP2 immunohistochemical staining at 20X magnification. (B) Quantification of the Ashcroft score, Masson’s trichrome positive area, and STEAP2 positive area across two groups. (C) RT‐qPCR assays to measure STEAP2 expression (n = 4 per group). (D) Western blot analysis to detect STEAP2 protein levels. Significance levels are indicated as *p < 0.05; **p < 0.01.

Subsequent to the bioinformatic analysis of IPF clinical patients' sequencing data, we proceeded to the in vivo study to investigate key regulators associated in Cu metabolism. IPF mouse model was constructed by intratracheal administration of bleomycin (5 mg/kg) to induce pulmonary fibrosis. After euthanasia, the left lung of IPF mouses was removed for H&E, Masson and STEAP2 immunohistochemical staining (Figure 5A). In comparison to the control group, the IPF group exhibited a range of inflammatory cell infiltration, accompanied by minor haemorrhage, alveolar wall thickening, an increased presence of collagenous fibres, and an increase in STEAP2 expression. In addition, we used right lung tissue for RT‐qPCR and Western Blot detection. The mRNA expression of STEAP2 showed an increased in the IPF group (Figure 5C), which was paralleled by a substantial increase in its protein expression, depicted in Figure 5D.

### 3.6 Knockdown of STEAP2 alleviates pulmonary fibrosis

**FIGURE 6:**
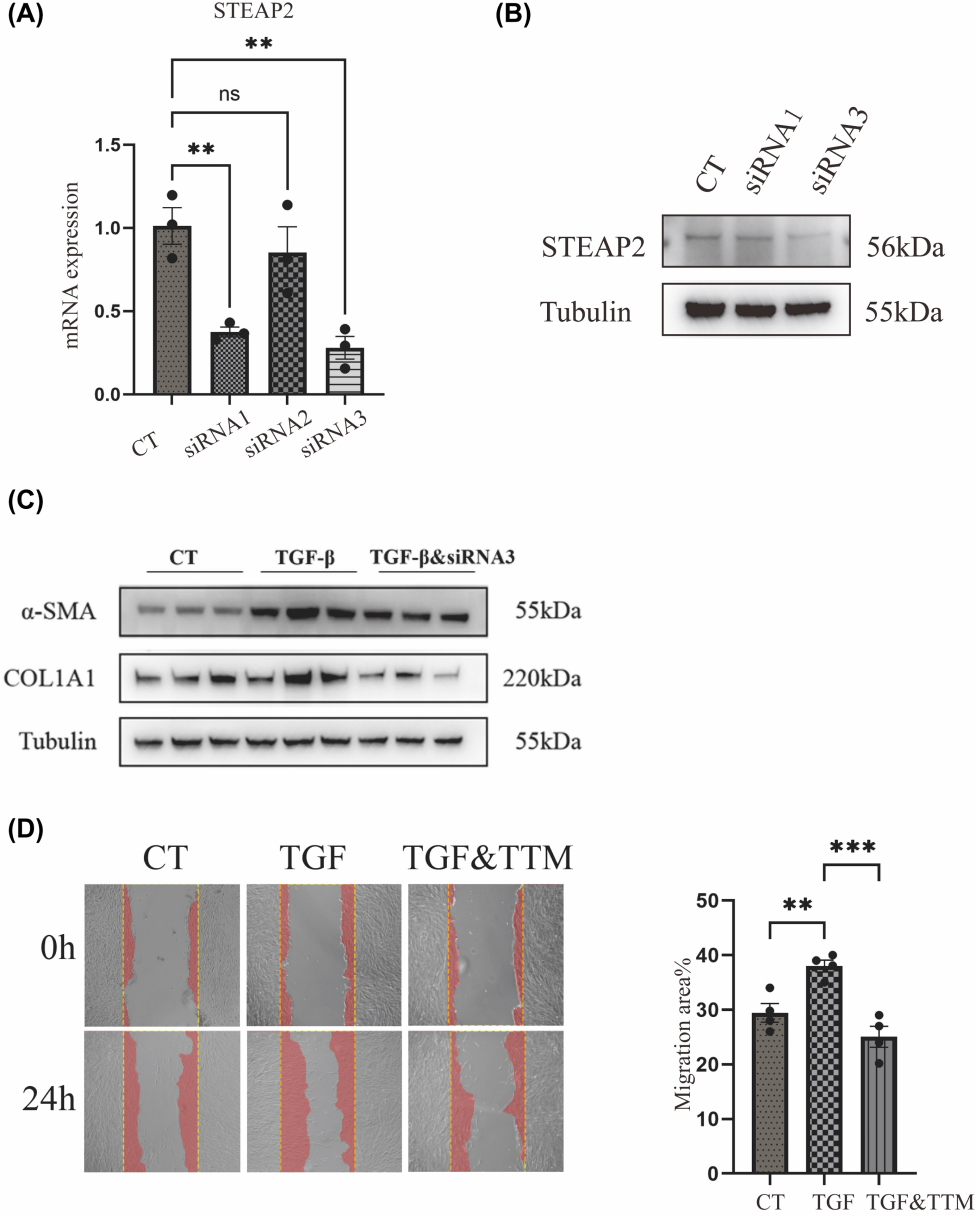
STEAP2 Inhibition. (A) RT‐qPCR assays quantifying STEAP2 expression in siRNA groups compared to the control (CT) group. (B) Western blot analysis of STEAP2 expression in the CT group and two siRNA groups (siRNA1 and siRNA3). (C) Western blot analysis measuring α‐SMA and COL1A1 levels across the three groups. (D) Scratch assays conducted over 24 hours to assess cellular migration in the three groups.

siRNA targeted STEAP2 was transfected into MRC5 cells to knock down its expression. RT‐qPCR experiments revealed notable suppression of STEAP2 expression with both siRNA1 and siRNA3, as depicted in Figure 6A. To further validate these findings, we performed Western Blot analyses which confirmed that siRNA3 significantly inhibited STEAP2 expression (Figure 6B). siRNA3 transfected cells showed significant resistance to TGF‐β induced fibrosis markers (α‐SMA and COL1A1, Figure 6C). Cell migration is also an important observation indicator for the differentiation of fibroblasts into myofibroblasts. It was found that after the addition of TGF‐β, the area of cell migration increased significantly from an average of 29%–37%. However, after the knockdown of STEAP2, cell migration was significantly inhibited, decreasing to 23% (Figure 6D). Therefore, we conclude that inhibiting the expression of STEAP2 contributes to the improvement of fibrosis.

## 4 DISCUSSION

IPF is a chronic, lethal and progressive pulmonary disease that contributed to significant morbidity and mortality. The clinical presentations of IPF varies, with progressive cough and dyspnoea being the most prevalent ones.^23^, ^24^ The rising prevalence of IPF has exerted a notable influence on both the economic progress of human society and the physical health of individuals.^25^ Current pharmaceuticals research can only mitigate the advancement of the disease and preserve lung function rather than offer a cure.^26^ The recently reported programmed cell death pathway, Cuproptosis, has taken the spotlight of oncological studies, where inducing tumour cell death by overloading its intracellular Cu concentration would become a potential therapeutic approach. However, researches of Cuproptosis in the pathogenesis and pathophysiological mechanism of inflammatory diseases haven't been abundant. Cu‐dependent cell death or Cuproptosis was implicated in disease progression, marked by the aggregation of lipoylated mitochondrial enzymes and the destabilization of iron–sulfur cluster proteins.^8^ It was reported that Cuproptosis was related to various diseases, such as rheumatoid arthritis,^27^ Crohn's disease,^28^ melanoma and lung adenocarcinoma.^29^ Interestingly, increased Cu concentrations in the bronchoalveolar lavage fluid (BALF) of IPF patients have been reported,^30^ with Cu‐dependent enzymes like Lysyl Oxidase‐Like 2 (LOXL2) implicated in the pathogenesis of pulmonary fibrosis.^31^

Previous studies have primarily relied on bioinformatic analyses to identify differentially expressed CRGs between IPF and healthy samples. While such studies offer valuable insights for future research directions, the absence of experimental validation limits their generalizability and applicability. In our study, we used multiple GEO datasets to minimize sample errors and enhance credibility. Prior to merging three datasets, we meticulously selected samples that met the standard diagnostic criteria for IPF. Our investigation focused on elucidating the relationship between CRGs, Cu metabolism‐related genes, and DEGs, leading to the identification of 14 shared DEGs associated with Cu metabolism. Subsequently, we examined the correlation between immune cell infiltration and Cu metabolism‐related DEGs. Furthermore, hub genes were examined in IPF samples, ultimately revealing STEAP2 as significantly upregulated genes in IPF, which also had an inverse correlation to resting NK cells levels, and they were significantly reduced in IPF samples compared with the control samples. We validated our findings using an animal model, wherein the manifestations observed in the mouse IPF model corroborated with the findings from human sequencing expression profiling. Finally, knockdown of STEAP2 conferred protection to MRC5 cells against TGF‐β induced fibrosis. By combining bioinformatic analyses with experimental validation and animal models, our study contributes to a deeper understanding of IPF pathogenesis and highlights STEAP2 as a potential therapeutic target.

STEAP2 is a member of the six transmembrane epithelial antigen of the prostate, and is predominately involved in vital physiological process such as ion transport and redox reactions.^14^ STEAP2 acts as a metalloreductase, facilitating the reduction of metal ions like ion and Cu.^32^ STEAP2 is widely expressed in various tissues and cell types, including lungs, kidneys, and immune cells, which suggests the potential roles of STEAP2 in diverse physiological processes beyond its initially identified association with prostate cancer. Zhu et al. identified that the m6A modification of STEAP2 inhibits thyroid cancer progression.^33^ Zhang et al. revealed that STEAP2 promotes osteosarcoma progression.^34^ With the newly discovered Cuproptosis as a novel programmed cell death pathway stimulated by intracellular Cu overload, it's of vital importance to consider the roles STEAP2 may play in maintaining intracellular Cu homeostasis. From the perspective of Cu metabolism, STEAP2 reduces Cu^2+^ to its more active form, Cu^1+^, while also facilitating Cu uptake. We therefore hypothesize that the upregulation of STEAP2 in IPF patients contributes to the intracellular accumulation of Cu and eventually triggers Cuproptosis, resulting in continuous lung damage. Our study confirmed that knockdown of STEAP2 using siRNA can protect MRC5 cells against TGF‐β induced fibrotic responses, suggesting the potential application of STEAP2 as a therapeutic target.

Our study has certain limitations. Firstly, we did not conduct in vivo study to confirm the efficacy of STEAP2 knockdown in alleviating Bleomycin‐induced pulmonary fibrosis. Secondly, our assessment of decreased fibrotic markers was limited to immunoblot analysis to substantiate the potential of STEAP2 knockdown. However, despite these limitations, our study bears sufficient novelty by being among the pioneering investigations to successfully identify a marker related to Cu metabolism and affirm its role in fibrotic damage. We believe that our findings underscore the importance of further research focusing on elucidating the mechanisms of Cu metabolism in the pathophysiological responses of IPF. More spotlights should be cast to fully elucidate the Cu metabolism mechanisms in pathophysiological responses of IPF in order to discover and develop novel therapeutic applications.

## 5 CONCLUSION

In conclusion, this comprehensive discussion elucidates the intricate molecular landscape of IPF, emphasizing the significance of DEGs, Cu metabolism, immune cell dynamics, and the pivotal role of STEAP2 in the disease process. These findings not only deepen our understanding of the pathogenesis of IPF but also provide a basis for future research and potential therapeutic interventions targeting STEAP2 and associated pathways. The integration of multi‐omics data and experimental validation enhances the credibility and translational relevance of our results, contributing to the ongoing efforts to unravel the complexities of IPF.

## AUTHOR CONTRIBUTIONS

**Yajun Wang:** Data curation (lead); formal analysis (lead); investigation (equal); visualization (equal); writing – original draft (lead). **Shuyang Chen:** Data curation (equal); formal analysis (equal); methodology (equal); writing – review and editing (lead). **Shujing Chen:** Conceptualization (equal); funding acquisition (equal); supervision (supporting). **Jinjun Jiang:** Conceptualization (equal); funding acquisition (equal); supervision (equal).

## CONFLICT OF INTEREST STATEMENT

The authors declare that the research was conducted in the absence of any commercial or financial relationships that could be construed as a potential conflict of interest.

## Data Availability

The data that support the findings of this study are available in NCBI at www.s4trials.com, reference number [GSE110147, GSE24206, GSE53845].
